# Flash-induced nanowelding of silver nanowire networks for transparent stretchable electrochromic devices

**DOI:** 10.1038/s41598-018-20368-3

**Published:** 2018-02-09

**Authors:** Chihak Lee, Youngsu Oh, In Seon Yoon, Sun Hong Kim, Byeong-Kwon Ju, Jae-Min Hong

**Affiliations:** 1 Photo-Electronic Hybrids Research Center, Korea Institute of Science and Technology (KIST), Seoul, 02792 Republic of Korea; 2 Display and Nanosystem Laboratory, College of Engineering, Korea University, Seoul, 02841 Republic of Korea; 3 Division of Nano & Information Technology, KIST School, Korea University of Science and Technology, Seoul, 02792 Republic of Korea; 4 Institute of Advanced Composite Materials, Korea Institute of Science and Technology, Jeonbuk, 55324 Republic of Korea

## Abstract

Electrochromic devices (ECDs) are emerging as a novel technology for various applications like commercialized smart window glasses, and auto-dimming rear-view mirrors. Recently, the development of low-power, lightweight, flexible, and stretchable devices has been accelerated to meet the growing demand in the new wearable devices market. Silver nanowires (AgNWs) can become new primary transparent conducting electrode (TCE) materials to replace indium tin oxide (ITO) for ECDs. However, issues such as substrate adhesion, delamination, and higher resistance still exist with AgNWs. Herein, we report a high-performance stretchable flash-induced AgNW-network-based TCE on surface-treated polydimethylsiloxane (PDMS) substrates. A Xe flash light method was used to create nanowelded networks of AgNWs. Surface silane treatments increased the adhesion and durability of the films as well. Finally, ECDs were fabricated under the optimal conditions and examined under strained conditions to demonstrate the resistance and mechanical behaviours of the devices. Results showed a flexible and durable film maintaining a high level of conductivity and reversible resistance behaviour, beyond those currently achievable with standard ITO/PET flexible TCEs.

## Introduction

Electrochromic devices (ECDs) are changing the way optical properties (e.g. reflectance, transmittance, and absorbance) can be controlled through an applied voltage. This has triggered interest and research on ECDs, shedding light on potential applications including smart windows, auto-dimming rear-view mirrors, sunglasses, and flexible displays^1–5^. Additionally, ECDs have proven a natural fit with wearable electronics due to their lower power consumption and better flexibility than traditional rigid glass-substrate devices^6–8^. However, scalability issues remain a concern for fully optimized, low-cost, and marketable ECDs due to restrictions on current transparent conductive electrode (TCE) materials relative to functionality and durability.

A wide variety of inorganic compounds like tungsten oxide (WO_3_), phthalocyanines (Lu(Pc)s), vanadium oxide (V_2_O_5_), and cerium-titanium oxide (CeO_2_TiO_2_) have been explored for use in ECDs^9–13^. While these inorganic compounds show strong pigmentation, processing them for large-area flexible instances is difficult due to their inherent brittleness and high production costs. Thus, a low-cost organic alternative would be better suited to respond to the increasing demands for ECD materials. Well established work has already been carried out in this field, with specific advances in the use of electrochromic polymers and hydrogels for ECDs^14^.

The field of TCEs for use in ECDs is still relatively nascent, and thus provides multiple opportunities for exploring novel materials. This is particularly important for the overall construction of flexible ECDs because the TCE component is typically the most expensive and has the highest impact on the overall ECD performance^15^. Indium tin oxide (ITO), fluorine-doped tin oxide (FTO), PEDOT:PSS, metal nanowires, graphene, carbon nanotube are some of the most commonly used TCE materials^16–18^. Amongst these, ITO has been the most popular due to its excellent optical transparency and conductive properties. However, the demand for alternative materials to ITO has escalated because low abundance of raw indium, high production and processing costs, and the brittle nature of ITO do not make it a strong option for stretchable ECDs^19,20^. Currently, silver nanowires (AgNWs) are the most promising candidate for an alternative TCE material as they have comparable optical and electrical properties seen in conventional ITO TCEs^20–24^. However, there are intrinsic weaknesses regarding the resistance and adhesion properties of AgNWs that need to be overcome for full utilization as transparent electrodes for ECDs.

Light-induced nanowelding operates via highly localized heating of NW junctions, and is particularly useful for fabricating AgNW networks that facilitate atomic mass transport and conduction behaviour^25–29^. The resistance of these NW junctions after nanowelding is dramatically decreased compared to that of the unirradiated junctions. From a processing stand point, light-induced nanowelding has limited application for mass production due to the time-consuming serial nature of the technique^28,29^. A possible alternative methodology for decreasing AgNW resistance through similar mechanisms can be seen in xenon flash lamp processing. This processing offers a broad-spectrum light source and opportunities for higher output efficiency both in terms of process speed and larger process areas^30^. This has made it an attractive option in the field of NW networks and has shown compatibility with roll-to-roll manufacturing^25–27^.

Another issue concerning the application of AgNWs to TCEs is the interfacial adhesion between the electrode and the substrate^31,32^. Delamination of the conductive material from the polymer substrate can seriously degrade the device’s reliability, functionality, and performance^33^. Methods to ensure better adhesion, such as application of higher pressure^34,35^, *in-situ* polymerization^23^, and encapsulation^21^ have been reported to be promising. Surface-modified polymer substrate techniques present the most viable option because they combine stronger adhesion properties with a high degree of flexibility for use in device substrates.

Herein, the development of an AgNW-based TCE is presented as a means to combine these positive attributes for the replacement of conventional ITO/PET components for electrochromic devices. To overcome adhesion between the hydrophilic AgNWs and hydrophobic PDMS substrates, a high-density coordination-type bonding process was utilized. In addition, flash-light plasmonic sintering techniques were applied for the TCE fabrication, resulting in substantial reduction in the device contact resistance. Endurance bending tests comparing AgNWs and ITO/PET TCE materials showed superior longevity and lower degradation under both iterative and singular strains. To investigate any trade-off between the transmittance and the sheet resistance of the AgNW network relative to its weight ratio, further analysis was performed to optimize the mass and energy densities of the AgNWs by comparing their figure of merit (FoM) values. Thus, we successfully fabricated stretchable TCEs for use in ECDs, and identified the respective switching times (t_color_/t_bleach_) for the ECDs under strained and unstrained conditions. Here t_color_ represents the time taken for the ECD to enter a low transmittance state, and t_bleach_ represents the time for the ECD to return to its original transparent state when no external voltage is applied. The techniques proposed here show large-scale applicability for patterning ECDs and the advantages in using flexible materials in place of fracture-prone ITO modalities.

## Results and Discussion

**Figure 1:**
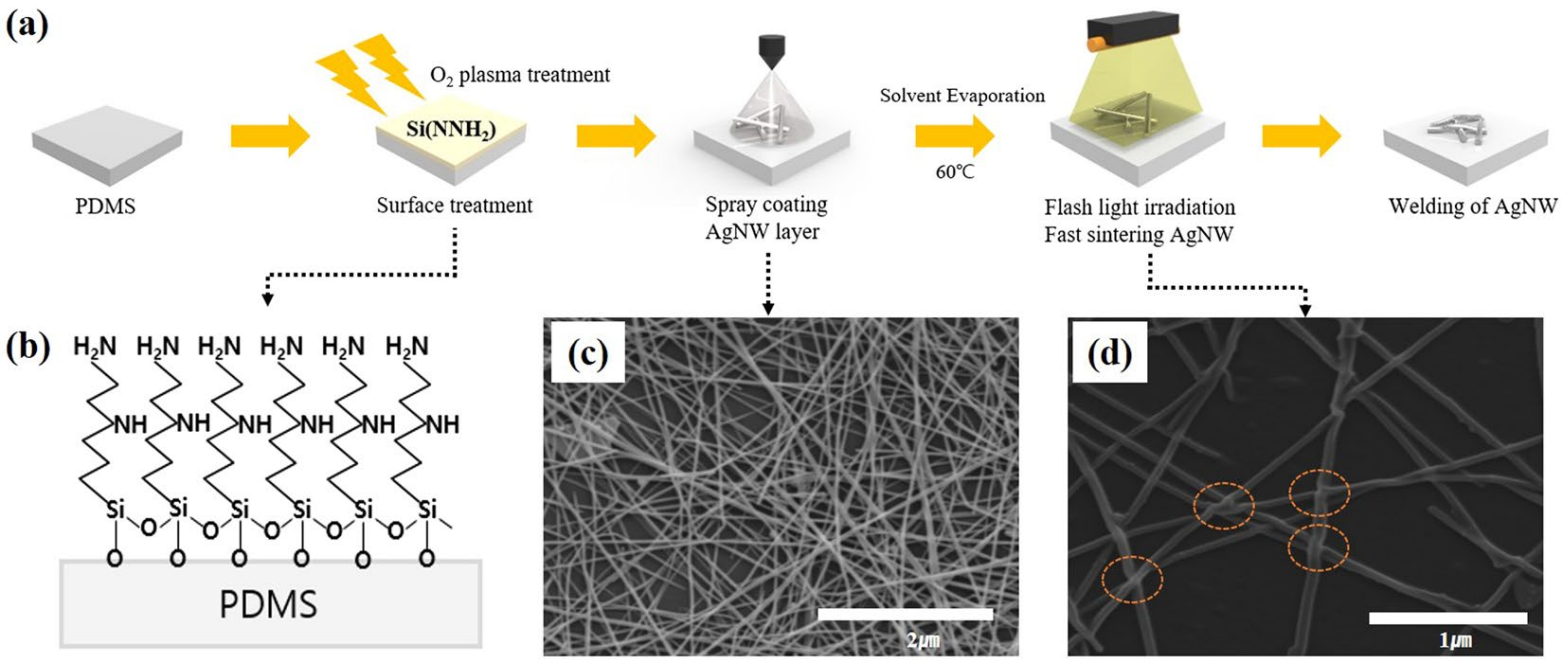
(**a**) Schematic diagrams of the fabrication process of the AgNW TCEs. (**b**) Surface plasma treatment to generate exposed hydroxyl groups, followed by immersion in a Si (NNH_2_) solution. SEM images of AgNW TCEs on silanized PDMS: (**c**) 0.4 wt % pristine AgNW, and (**d**) flash light induced AgNW TCEs.

The fabrication process for the stretchable AgNW TCEs is represented in Fig. 1(a). Three states were investigated for their respective roles in the fabrication process: surface treatment, AgNW stacking, and flashlight-induced nanowelding. The first phase of the fabrication consisted of a surface treatment of the PDMS substrates with an O_2_ plasma to develop exposed surface hydroxyl groups, and was subsequently followed by immersion in Si(NNH_2_) and 10% ethanol solution. Silane molecules with the NNH_2_ head groups, shown in Fig. 1(b), were created to control the interactions between the AgNW dispersion and the functional head groups present on the substrate; this serves to promote stronger hydrophilicity. AgNW inks (0.4 wt% AgNW, Nanopyxis CO, Ltd Korea) were then spray-coated onto the pre-treated PDMS surfaces to form AgNW networks with high NW junction resistance. A more detailed explanation regarding the processing and fabrication steps can be found in the methods section at the end of this work. Characterization of the AgNW TCEs varying morphologies were characterized via scanning electron microscope (SEM) and are further illustrated in Fig. 1(c). Results of this showed that initial networks of AgNWs were loosely tangled. AgNW networks were then irradiated using the xenon flash light method (pulse duration of 10 ms), which nanowelded the AgNW junctions. SEM imaging of this nanowelding process is shown in Fig. 1(d).

**Figure 2:**
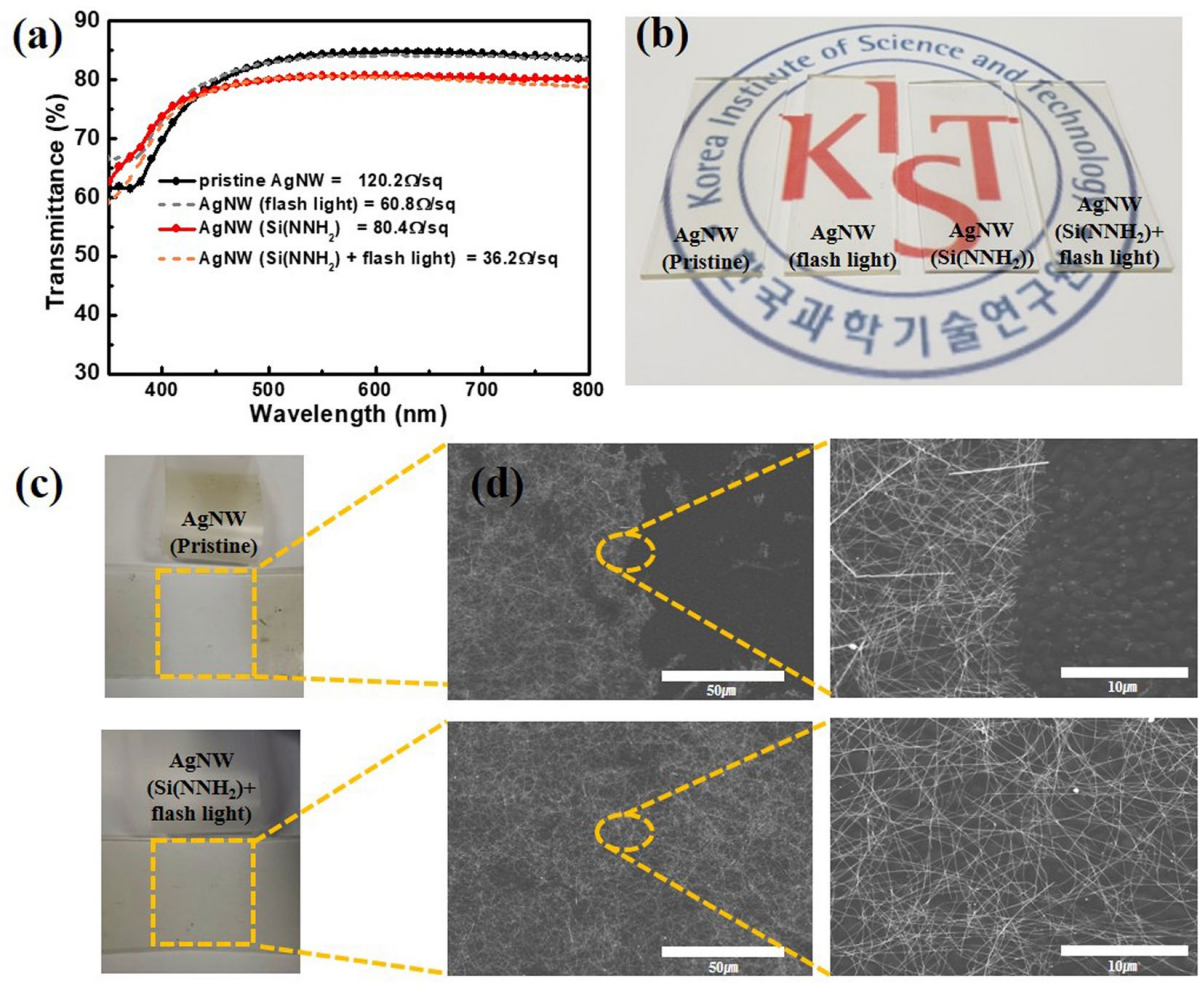
(**a**) Transmittance spectrum of the various samples and relative photographs (**b**) Photograph comparing surface treated, and non-treated pristine AgNW TCEs with and without flash light exposure. (**c**) Photographs of the pristine AgNW and treated AgNW TCEs after the Scotch tape adhesion test. (**d**) SEM images of the pristine AgNW and treated AgNW TCEs after the adhesion tests.

As shown of Fig. 2(a), comparisons were made on AgNW TCEs according to transmittance and sheet resistance, surface treatment, and adhesion behaviour. In the first assessment of transmittance and sheet resistance little variation was found in the transmittance of the films when exposed to the flash treatment versus not being exposed. However, sheet resistance values showed an approximate 50% decrease between the flash-treated and the unaltered surfaces. This provides evidence which suggests the success of the nanowelding technique at joining the overlapping AgNWs. A second assessment relative to the surface silanization was carried out and showed similar results, but based on different mechanisms. Here, transmittance was only marginally affected by the silane surface treatment (~3%), but sheet resistance was shown a reduction of ~30% because of improved contact and bonding to the PDMS substrate. Thus, the findings of these two results suggest that the application of the surface treatment coupled with the flash light technique yielded optimal properties given the bounds of this experiment. The last assessment of these properties was carried out to investigate the adhesion between the pristine AgNW TCEs and the surface-treated AgNW TCEs. This was carried out via a Scotch tape adhesion test, the results of which are shown in Fig. 2(c). Non-treated AgNW TCEs showed the greatest instance of delamination regions along the substrate surfaces, in contrast to the silane-treated substrates that remained largely intact. This can be attributed to the success of the silanized PDMS in promoting higher hydrophilic character to match the behaviour of the AgNW-isopropanol dispersion. The result is a strong bond between the AgNWs and the PDMS and is reflected here by a reduction in delamination events during the Scotch tape adhesion tests.

**Figure 3:**
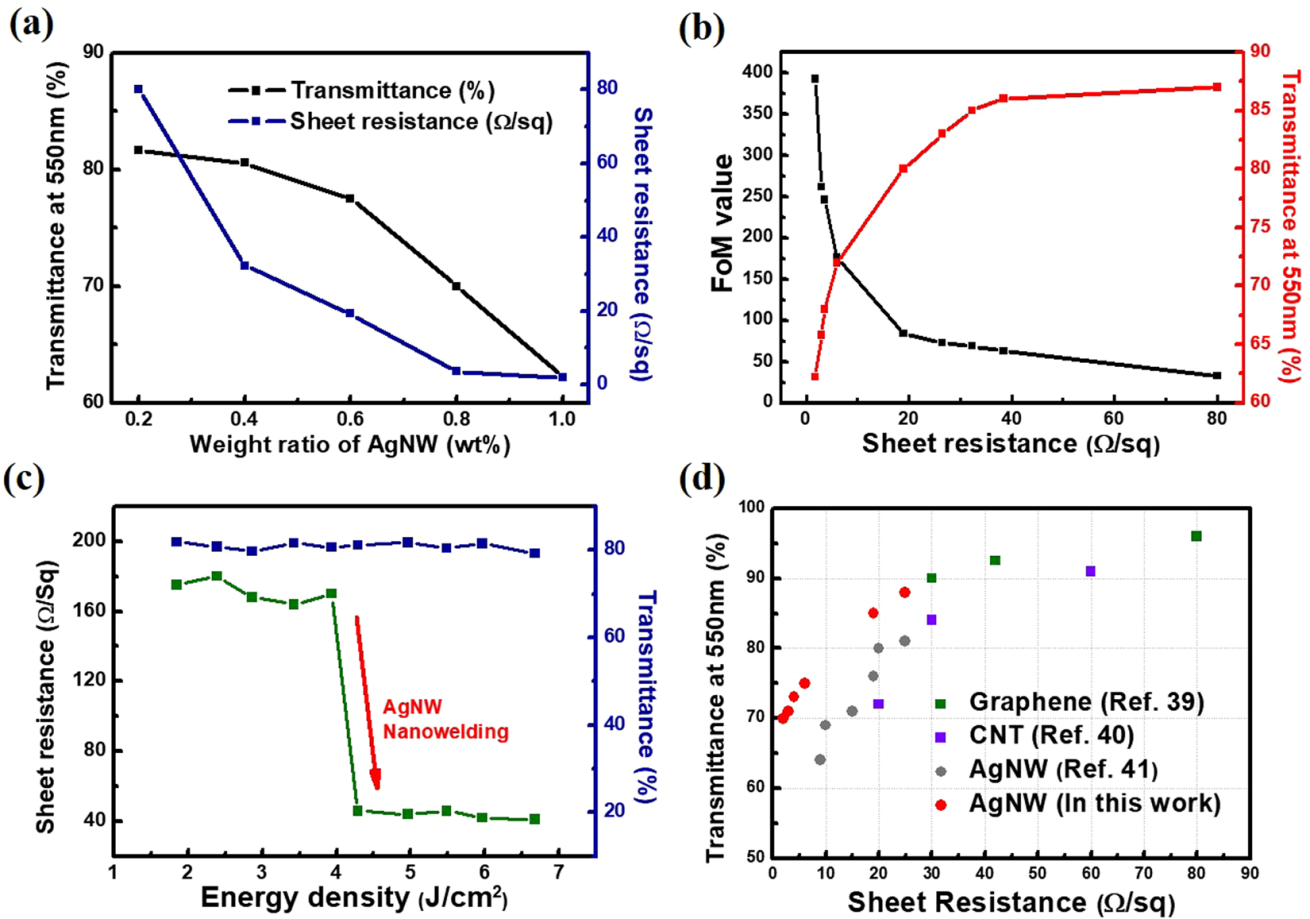
(**a**) Transmittance and sheet resistance values relative to the weight ratio of AgNW. (**b**) Transmittance (at λ = 550 nm) and Tinkham equation figure of merit (FoM) values as a function of the sample sheet resistances. (**c**) Sheet resistance and transmittance response to the flash light method induced nanowelding. (**d**) Resulting sheet resistance and transmittance values compared to reference values from literature.

The sheet resistance and transmittance of the AgNW TCEs were shown to be related to both the NW concentration and the flash light energy density. To determine this relation the optimum AgNW concentration was found for the fabrication of AgNW TCEs by varying the AgNW-isopropanol solutions to include the five different weight percent values shown in Fig. 3(a). The results showed that the sheet resistance of the AgNW TCEs was improved relative to increasing AgNW density, and a corresponding decrease in transmittance was also observed. The FoM concept was used here to define the best-optimized conditions for TCE fabrication by taking into account the trade-off between sheet resistance reduction and transmittance^36^. This relationship is expressed in the equation below between transmittance (σ) and sheet resistance (R) for a given TCE.

1 $$T({\lambda })={\{1+\frac{{R}_{0}}{2{R}_{s}}\frac{{\sigma }_{OP}({\lambda })}{{\sigma }_{DC}}\}}^{-2}$$

Equation 1. Figure of merit relationship between sheet resistance and optical transmittance at 550 nm.

Here, σ_op_ (λ) represents the optical conductivity (at 550 nm) and σ_DC_ is the direct current (DC) conductivity of the TCEs^22,37^. The σ_op_/σ_DC_ value is used as a FoM for an eidetic comparison of the sheet resistance and transmittance expressed in Fig. 3(b). This plot shows that all calculated FoM values (σ_op_/σ_DC_) for the AgNW TCEs were >35 and thus show superior behaviour to the standard values observed in industrial applications^37,38^. In Fig. 3(c), the sheet resistance and transmittance are expressed as a comparison to varying flash-light energy densities. When the flash light pulsing interval was held constant at 10 ms, irradiation energies greater than 4 J/cm^2^ considerably reduced the AgNW TCEs sheet resistance in every instance. No such substantial behaviour was found when testing for the dependence of irradiation energy on transmittance. This provides support for similar, and even improved, property enhancements for AgNW TCEs when compared against longer time-consuming furnace heat treatment processes. Additional improvements to the AgNW sheet resistances were not seen at energies higher than 4 J/cm^2^. This is attributed to the nanowelds which exist in a more binary state. In other words, once the welds are established at a certain threshold energy, any additional energy supplied to the system does not increase the degree to which the NWs are welded together along the NW network. Figure S1 goes into greater detail with this observation and shows that excessive irradiation above this optimized energy threshold can actually degrade or even break the AgNW network. In Fig. 3(d) a broader comparison was carried out relative to the electrical and optical properties observed here and in previous reports. Comparing these methodologies shows that the flash light induced AgNW method performs well in comparison to other existing techniques such as; graphene^39^, carbon nanotubes (CNTs)^40^, and reference unwelded AgNWs^41^.

**Figure 4:**
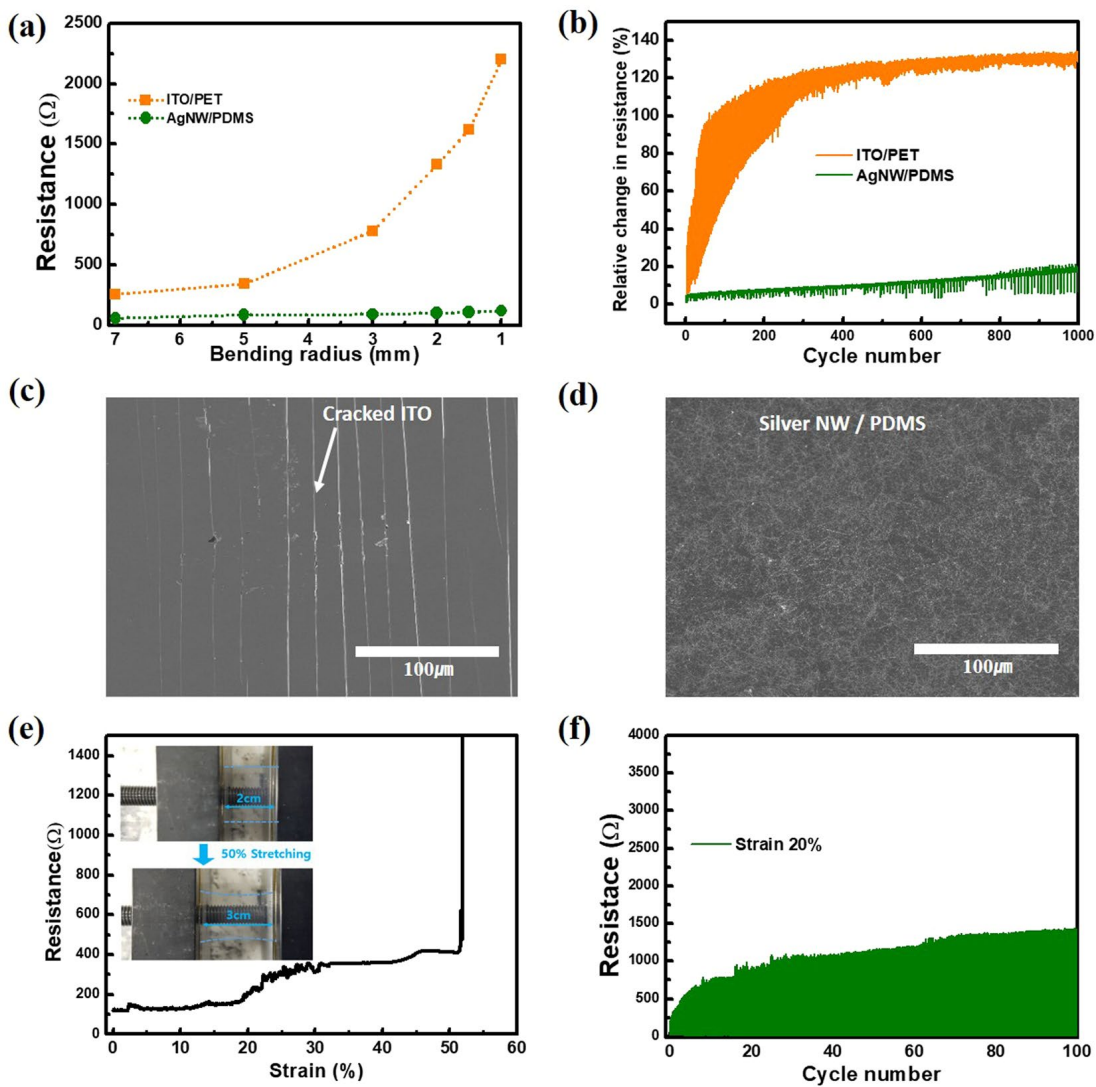
(**a**) Resistance response of increasing bending radius (r_b_) between ITO/PET and AgNW based TCEs. (**b**) Resistance response relative to number of bending cycles (at a constant r_b_ = 2 mm) underwent by the ITO/PET and AgNW based TCEs. (**c**) FE-SEM images showing the crack propagation pattern in the ITO/PET film after 1000 cycles, and (**d**) showing the AgNW TCEs under the same parameters. (**e**) Stretching performance relative to resistance as a function of stretching induced strain (%); the inset shows a photograph of this stretching on the AgNW TCEs. (**f**) Cycle performance of a representative AgNW TCEs under stretching 20% strain.

To compare the mechanical durability of the AgNW TCEs against the flexible ITO/PET bending tests were performed at varying bending radius (r_b_) values. Figure 4(a,b) illustrates the resistance and the change in resistance according to the radius of curvature of the given films. Here, the change in resistance is represented by R/R_0_, where R is curved state resistance and R_0_ is the flat state resistance. The AgNW TCEs were shown to have no considerable long-term change in R_s_ when a corresponding change in r_b_ was implemented. In contrast to ITO/PET films which showed gradual degradation in R_s_ values when the r_b_ was increased. This is because the ITO was deposited as an additive thin film which results in a brittle oxide layer, compared to the AgNW network which is bound to the surface and has a less bulk-like structure that allows for more mechanical motion of the layer. Additionally, any resistance shift in the AgNW TCEs induced by the bending was recovered once the film was released and allowed to return to its original shape. The damage incurred from the bending on the ITO/PET samples, however, was not shown to be reversible; examples of which can be seen in Fig. 4(b). SEM observations confirm the existing understanding that ITO/PET is a relatively brittle and weak material, with damage persisting after bending events. Direct observations of the AgNW films show minimal latent damage under similar circumstances and indicate greater durability than that for the ITO/PET systems. Figure 4(e) shows the stretchable AgNW TCEs behaviour under various test strains. This confirmed that while the PDMS substrates are highly stretchable, their strong hydrophobic nature is incompatible with the hydrophilic AgNWs. This confirms the need for the surface treatment discussed earlier and the results shown in the 20% strain cycling tests of Fig. 4(f) also support this notion. From this data, the maximum strain before fracture was found to be ~50% with a corresponding R/R_0_ ratio of approximately 4. A gradually degraded value for R/R_0_ was observed over repeated strains, but the film always recovered any additional increase in resistance brought on by the initial stretching. This speaks to the qualities of the AgNW network and PDMS working in tandem to produce an elastic and conductive material.

**Figure 5:**
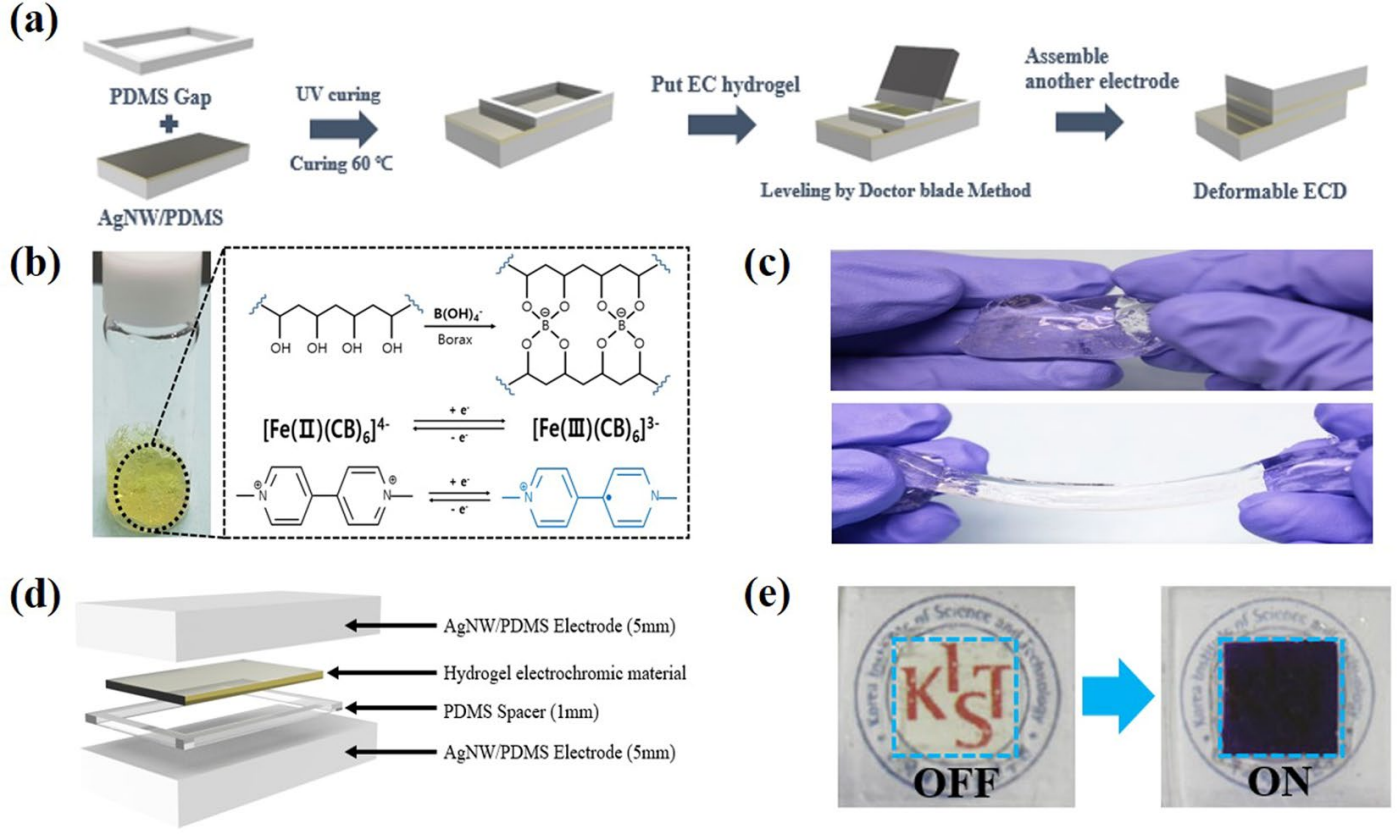
(**a**) The fabrication process of stretchable ECDs using AgNW TCEs. (**b**) Material composition of the stretchable electrochromic material. (**c**) A demonstration of the stretching performance of the PVA-Borax based hydrogel. (**d**) Schematic illustrating the stretchable ECD stacking. (**e**) ON/OFF response of the ECDs; ON = −2.3 V applied and OFF = 0 V applied.

With the results and findings from these assessments, stretchable ECDs were fabricated using the AgNW TCEs. This is shown schematically in Fig. 5(a) where top and bottom AgNW TCEs and PDMS spacers are separated with an electrochromic hydrogel. A more detailed explanation of the experimental steps taken to produce these ECDs is discussed in the methods sections. A variation of this structure is shown in Fig. 5(b), where the electrochromic hydrogel was made with the combination of a PVA-borax hydrogel and ethyl viologen. Examination of this material is shown in Fig. 5(c) with results indicating that the PVA-borax hydrogel possesses strong elastic characteristics. These property provide additional benefits for stretchable ECDs since a capacity for elastic behaviour is highly advantageous in systems where regular film deformation occurs. However, depending on the filler or the hydrogel material used for the specific industrial purpose, these effects may not be observed because many solid copolymer electrochromic materials lack this ability to overcome mild deformations. Liquid type electrochromic materials do exist and have been applied in stretchable ECD scenarios, but are prone to leaking and present additional processing challenges involving the encapsulation of the filler material. Thus, current findings suggest that hydrogel type models are the most suitable for stretchable ECDs. The PDMS spacers play an important role on the performance of ECDs through the thickness of the PDMS affecting the transmittance. A schematic of such a film is shown in Fig. 5(d). These effects can be mitigated through patterning of the PDMS, which presents an area for further optimization of future ECDs. Figure 5(e) shows a photograph of a normal (1 cm by 1 cm) state ECD. Here it can be observed that following an applied voltage the ECD colour changes to purple and allows the ECD to block certain spectra of light.

**Figure 6:**
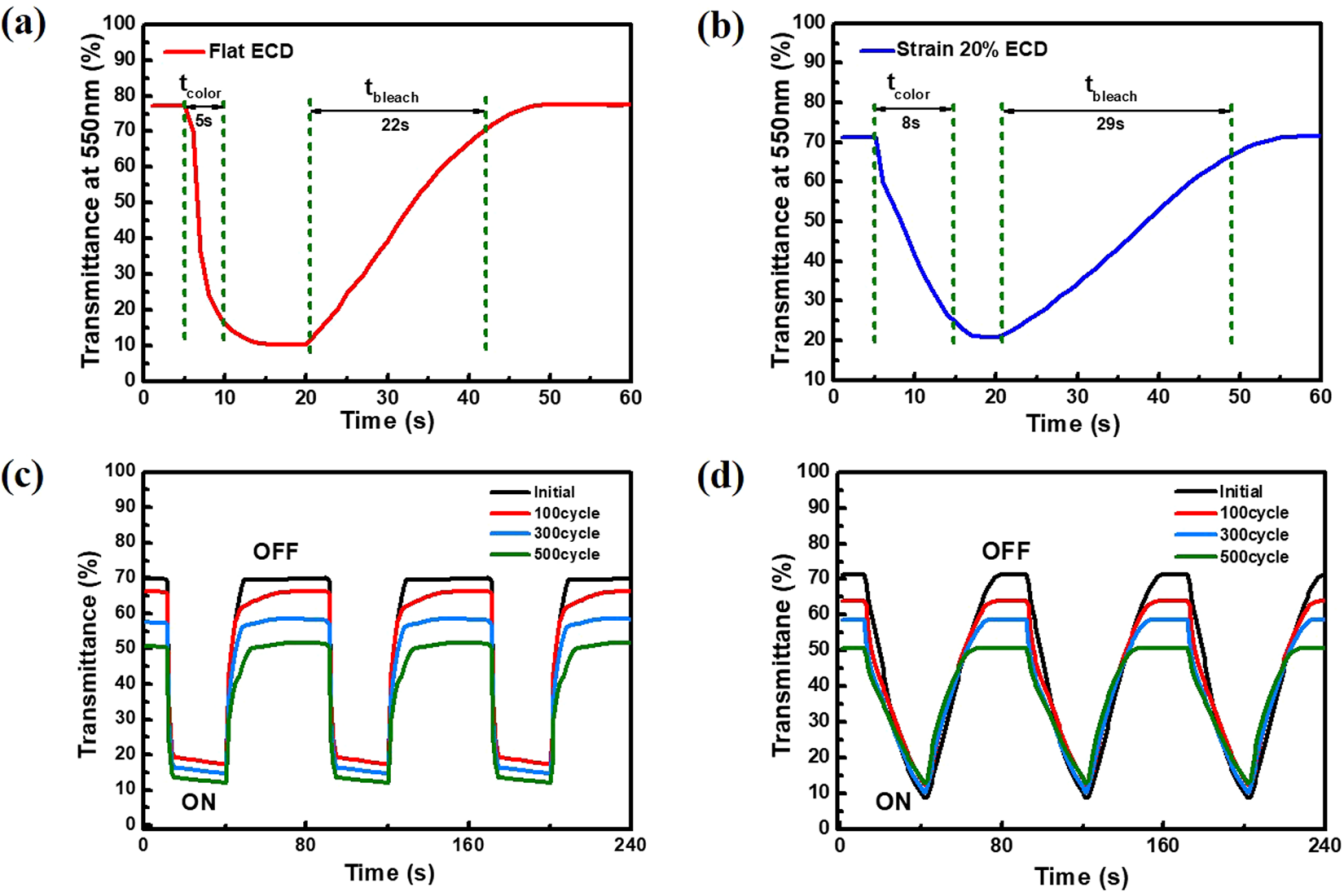
(**a**) Transmittance at 550 nm as a function of the time for the flexible ECDs in a flat state. Here specific regions show the transition to a coloured state (t_color_) and then a return to an uncoloured state (t_bleach_). (**b**) Similar transmittance data is shown for ECDs in a bent state. (**c**) The ON/OFF cycle test of the ECDs in the flat state, and (**d**) for ECDs in the 20% strain state.

Lastly, switching times for ECDs were tested relative to their dependence on flat or stretched states. In figure S2, we demonstrated the flat ECD and 20% stretching ECD with 2.3 V voltage applied. Figure 6(a) shows that the flat ECDs have a switching time. We have defined t_color_ as the time when it becomes 90% of the color change and conversely the t_bleach_ is defined as 90% of the change which becomes transparent. At flat state, t_color_ = 5 s and t_bleach_ = 22 s. Under a stretched scenario with an applied strain of 20%, the switching time showed a proportional 20% larger value than that seen in the flat state, Fig. 6(b). This is because of the increasing strain dependent resistance of the electrochromic hydrogel; similar behaviour was seen for AgNW TCEs when compared from a flat to stretched state. Thus, findings suggest that the resistance of the ECD depends on the degree of strain or stretched state of the film. To test this dependency, our devices were subjected to repeated cycle tests, by alternating between an operating voltage of 2.3 V and an off state of 0 V for 30 s and 50 s, respectively. The two types of ECDs were then subjected to these cycles up to 100, 300, and 500 times. During these repeated cycling tests, visible degradation in terms of optical contrast was seen in the ECDs; this is attributed to the hydrogel degrading and not the films themselves. Because of this, the optical contrast shifted from an initial state of 52.5% to 48.8% at 100 cycles, 42.7% at 300 cycles, and 38.7% at 500 cycles. The full results of this test can be seen in Fig. 6(c). When repeated for the 20% strain state samples, similar results were observed with an initial state of 51.3% moving down to 47.2% at 100 cycles, 42.4% at 300 cycles, and 37.7% at 500 cycles, Fig. 6(d).

**Figure 7:**
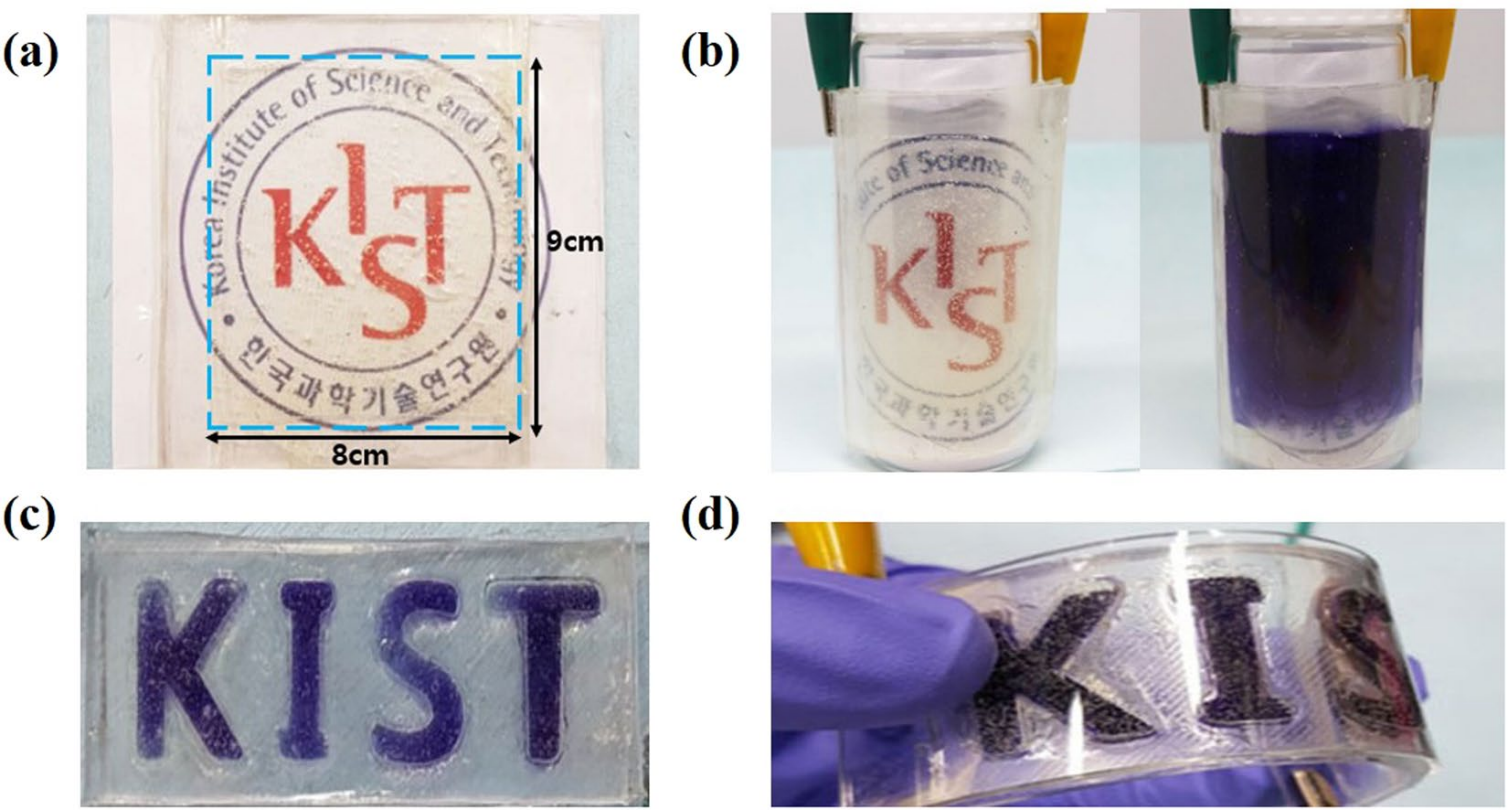
(**a**) AgNW TCE based ECDs were fabricated over a large area (8 cm × 9 cm) shown here (**b**) The flexibility of these ECDs is demonstrated via bending around a cylindrical vial (Φ = 5 cm and h = 10 cm) while in an ON state. (**c**) K, I, S, T letters patterned using ECDs and the spacer-doctor blade method (**d**) Showing the flexible performance of the patterned ECDs.

Figure 7(a) shows the larger AgNW TCE based stretchable ECDs that were tested in this work and their corresponding larger dimensions which would have been difficult to obtain using conventional nanowelding techniques (8 cm × 9 cm). In a more qualitative series of tests, ECDs were attached to a cylinder-shaped vial (Φ = 5 cm and h = 10 cm), and showed strong operational behaviour in this high curvature state in Fig. 7(b). In Fig. 7(c), we patterned “K”, “I”, “S”, and “T” atop the stretchable ECDs using 3D-printed moulds. Printed shapes were then bent and flexed as seen in Fig. 7(d), and again showed strong operational behaviour.

In conclusion, we demonstrated the fabrication and characterization of highly transparent and stretchable AgNW based TCEs for use in ECDs by assembling a AgNW surface network using xenon flash light methods and silane surface treatments to encourage strong adhesion. It was shown that the AgNWs provided efficient conducting pathways, but when applied without the addition of the silane surface treatment suffered from poor bonding due to the hydrophilic nature of the NWs in argument with the hydrophobic nature of the PDMS substrate. However, once the treatment was applied the AgNWs showed no substantial effect on transmittance, but did show a reduction in ECD sheet resistance with the application of the nanowelding technique by ~50%. Further it was found that relationship between sheet resistance and transmittance varied according to both the AgNW weight ratio and flash light energy density. The AgNW network, was then directly compared to flexible ITO/PET films and showed enhanced durability and flexibility. In this process, the energy density of 4 J/cm^2^ acted as a threshold energy for nanowelding to occur; beyond this point, no additional reduction or change in sheet resistance was observed. Finally, with the optimized parameters found in this work we fabricated stretchable ECDs using the AgNW TCEs and PVA-borax hydrogel. In testing for switching times it was shown that the strained state had a direct effect and could be seen in values of t_color_ = 5 s, t_bleach_ = 18 s for unstrained, and t_color_ = 10 s and t_bleach_ = 39 s for the 20% strain state. This is because of resistance change within the electrochromic hydrogel and AgNW TCEs, although it was also shown that initial increases in resistance of the TCEs was reversible once the strain was removed. Thus, here we have demonstrated an alternative method to create a nanowelded AgNW network for use as TCEs in ECD applications. In doing this we feel that this research opens new opportunities and applications to hasten the technology being brought to market for applications in wearable devices, military instances, flexible displays, and smart robotics.

## Methods

### Material

Silicone kit (SYLGARD 184), silver nanowires (NANOPHSIS CO, Ltd Korea with an average diameter: 30 nm, length: 25 µm), and [3-(2-aminoethylamino) propyl] trimethoxysilane (NNH_2_) and ethyl viologen, potassium ferrocyanide, ferricyanide were purchased from Sigma-Aldrich, and used as received. PVA, sodium tetraborate were purchased from Alfa Aesar.

### Preparation of PDMS based TCEs

PDMS (SYLGARD 184, Dow corning) was mixed with base and curing agent in 10:1 weight ratio. PDMS substrates was then pretreated with 50-sccm O_2_ plasma for 60 s to generate free surface oxygen groups (UV-Ozone cleaner, YuilUV CO, Ltd, Korea). Then, this layer was covalently functionalized via incubation with 10% NNH_2_ Solutions in ethanol, and heated at 60 °C for 90 min. Following this procedure, substrates were dipped and washed with ethanol. AgNWs were then deposited on this silanized PDMS film via an AgNW suspension diluted in isopropyl alcohol to various concentrations (0.2~1.0 wt%), prior to which mixing was carried out using a vortex device for 2 min. The AgNW suspension with various concentrations were then spray-coated onto PDMS substrate atop a hot plate at 80 °C. We used air brush spray gun (IWATA NEO HP-BCN AIR BRUSH, nozzle size 0.5 mm). Spraying distance was fixed over 15 cm using a clamp.We periodically sprayed AgNW solution for every 3 seconds and rested for 7 seconds to evaporate solvent. If the solvent is not fully evaporated, the silver nanowire will aggregate and become opaque. We sprayed 20 ml AgNW suspension each TCEs. The AgNW film was then exposed to a xenon flash light (pulse duration of 10 ms) with optimized energy discussed in the above results (the end optimal value was found to be 4 J/cm^2^).

### Intense Pulsed Light System

A custom-made intense pulsed light system was used for the photonic sintering and used a primary xenon flash lamp as the source (PerkinElmer QXF, UK). A water cooling system worked to maintain the temperature stability of the lamp, and a motion controller adjusted the z-axis translation stage for proper exposure of the fabricated films. The intense pulse light from the xenon flash lamp had a broad spectrum of light from 400 to 1000 nm. Sintering conditions were controlled by various factors such as voltage, pulse duration, pulse numbers, and operating time. The irradiation energy was measured by a NOVA II laser power meter (OPHIR), and the conductivity measurements were performed using a four-point probe method.

### Preparation electrochromic hydrogel

A 4% solution of PVA was prepared by placing 4 g of PVA and 96 mL of distilled water in a stirred system at 90 °C for 2 h. Also, ethyl viologen dibromide was added to the PVA solution in 20 mmol L^−1^ and 0.8 mmol L^−1^ amounts, an additional solution of a 1:1 mixture of potassium ferrocyanide and ferricyanide salts was also added. The resulting mixture was then stirred until a homogeneous solution was obtained. Separately, a 4% solution of sodium tetraborate was similarly prepared. Last the sodium tetraborate aqueous solution and PVA solution were mixed in a 1:4 volumetric ratio by vigorous stirring with spatula until a gel was obtained.

### Electrochromic device Fabrication

The preparation of electrochromic devices based on the AgNW/PDMS hybrid electrode were prepared via the following steps. First, prepared AgNW/PDMS electrodes were placed as the lowest layer of the film. Next, another pre-treated and cured PDMS (20:1 wt%) frame was placed on the AgNW/PDMS electrode as a gap between the filler, and then allowed to cure at 80 °C for 30 min to allow complete adhesion. The Electrochromic hydrogel was then used as a gap filler and levelled using a doctor’s blade. The other AgNW/PDMS electrode was then placed on top.

### Optical, Electrical, and Mechanical Characterizations

Transmittance and absorption were measured by employing an ultraviolet (UV)-visible spectroscope (Optizen 3220uv, Mecasys). The sheet resistance measurement was carried out by a 4-point probe system (Keithley 2400, source meter). SEM image were obtained using an (Inspect F50, FEI, USA) Cyclic bending test was performed using a bending tester (custom-made) with a digital multimeter to indicate the real-time line resistance.

## Electronic supplementary material

Supplementary information

## References

[1] Baetens R, Jelle BP, Gustavsen A (2010). Properties, requirements and possibilities of smart windows for dynamic daylight and solar energy control in buildings: A state-of-the-art review. Sol. Energ. Mat. Sol. Cells.

[2] Mortimer RJ, Dyer AL, Reynolds JR (2006). Electrochromic organic and polymeric materials for display applications. Displays.

[3] Chandrasekhar, P., Zay, B. J., Cai, C., Chai, Y. & Lawrence, D. Matched-dual-polymer electrochromic lenses, using new cathodically coloring conducting polymers, with exceptional performance and incorporated into automated sunglasses. *J. Appl. Polym. Sci*. **131**, 10.1002/app.41043 (2014).

[4] Huang L-M (2012). Photovoltaic electrochromic device for solar cell module and self-powered smart glass applications. Sol. Energ. Mat. Sol. Cells.

[5] Dyer AL (2014). A vertically integrated solar-powered electrochromic window for energy efficient buildings. Adv. Mater..

[6] Liang L (2013). High-performance flexible electrochromic device based on facile semiconductor-to-metal transition realized by WO_3_.2H_2_O ultrathin nanosheets. Sci. Rep..

[7] Polat EO, Balci O, Kocabas C (2014). Graphene based flexible electrochromic devices. Sci Rep.

[8] Costa C, Pinheiro C, Henriques I, Laia CA (2012). Inkjet printing of sol-gel synthesized hydrated tungsten oxide nanoparticles for flexible electrochromic devices. ACS Appl. Mater. Interfaces.

[9] Ko JH (2012). Graphene-based electrochromic systems: the case of Prussian Blue nanoparticles on transparent graphene film. Chem. Commun..

[10] Lampert CM (1984). Electrochromic Materials and Devices for Energy-Efficient Windows. Sol. Energ. Mater..

[11] Şen P, Dumludağ F, Salih B, Özkaya AR, Bekaroğlu Ö (2011). Synthesis and electrochemical, electrochromic and electrical properties of novel s-triazine bridged trinuclear Zn(II), Cu(II) and Lu(III) and a tris double-decker Lu(III)phthalocyanines. Synth. Met..

[12] Talledo A, Granqvist CG (1995). Electrochromic vanadium–pentoxide–based films: Structural, electrochemical, and optical properties. J. Appl. Phys..

[13] Avellaneda CO, Pawlicka A (1998). Preparation of transparent CeO_2_-TiO coatings for electrochromic devices. Thin Solid Films.

[14] Alesanco Y (2015). Polyvinyl Alcohol-Borax Slime as Promising Polyelectrolyte for High-Performance, Easy-to-Make Electrochromic Devices. ChemElectroChem.

[15] Emmott CJM, Urbina A, Nelson J (2012). Environmental and economic assessment of ITO-free electrodes for organic solar cells. Sol. Energ. Mat. Sol. Cells.

[16] Hong W, Xu Y, Lu G, Li C, Shi G (2008). Transparent graphene/PEDOT–PSS composite films as counter electrodes of dye-sensitized solar cells. Electrochem. Commun..

[17] Kim YH (2011). Highly Conductive PEDOT:PSS Electrode with Optimized Solvent and Thermal Post-Treatment for ITO-Free Organic Solar Cells. Adv. Funct. Mater..

[18] Tait JG (2013). Spray coated high-conductivity PEDOT:PSS transparent electrodes for stretchable and mechanically-robust organic solar cells. Sol. Energ. Mat. Sol. Cells.

[19] Kang M-G, Joon Park H, Hyun Ahn S, Jay Guo L (2010). Transparent Cu nanowire mesh electrode on flexible substrates fabricated by transfer printing and its application in organic solar cells. Sol. Energ. Mat. Sol. Cells.

[20] Lee JY, Connor ST, Cui Y, Peumans P (2008). Solution-processed metal nanowire mesh transparent electrodes. Nano. Lett..

[21] Hu LB, Kim HS, Lee JY, Peumans P, Cui Y (2010). Scalable Coating and Properties of Transparent, Flexible, Silver Nanowire Electrodes. ACS Nano.

[22] De S (2009). Silver Nanowire Networks as Flexible, Transparent, Conducting Films: Extremely High DC to Optical Conductivity Ratios. ACS Nano.

[23] Zeng XY, Zhang QK, Yu RM, Lu CZ (2010). A new transparent conductor: silver nanowire film buried at the surface of a transparent polymer. Adv. Mater..

[24] Madaria AR, Kumar A, Ishikawa FN, Zhou C (2010). Uniform, highly conductive, and patterned transparent films of a percolating silver nanowire network on rigid and flexible substrates using a dry transfer technique. Nano Res..

[25] Garnett EC (2012). Self-limited plasmonic welding of silver nanowire junctions. Nat. Mater..

[26] Park, J. H. *et al*. Flash-Induced Self-Limited Plasmonic Welding of Silver Nanowire Network for Transparent Flexible Energy Harvester. *Adv. Mater*. **29**, 10.1002/adma.201603473 (2017). 10.1002/adma.201603473 27892631

[27] Jiu J (2012). Strongly adhesive and flexible transparent silver nanowire conductive films fabricated with a high-intensity pulsed light technique. J. Mater. Chem..

[28] Han S (2014). Fast plasmonic laser nanowelding for a Cu-nanowire percolation network for flexible transparent conductors and stretchable electronics. Adv. Mater..

[29] Spechler JA, Arnold CB (2012). Direct-write pulsed laser processed silver nanowire networks for transparent conducting electrodes. Appl. Phys. A.

[30] Oh Y (2017). Selective photonic sintering of Ag flakes embedded in silicone elastomers to fabricate stretchable conductors. J. Mater. Chem. C.

[31] Zhu R (2011). Fused Silver Nanowires with Metal Oxide Nanoparticles and Organic Polymers for Highly Transparent Conductors. ACS Nano.

[32] Kumar ABVK, Bae CW, Piao L, Kim SH (2013). Silver nanowire based flexible electrodes with improved properties: High conductivity, transparency, adhesion and low haze. Mater. Res. Bull..

[33] Son B (2014). Measurement and analysis of adhesion property of lithium-ion battery electrodes with SAICAS. ACS Appl. Mater. Interfaces.

[34] Gaynor W, Burkhard GF, McGehee MD, Peumans P (2011). Smooth nanowire/polymer composite transparent electrodes. Adv. Mater..

[35] Gaynor W, Lee JY, Peumans P (2010). Fully Solution-Processed Inverted Polymer Solar Cells with Laminated Nanowire Electrodes. ACS Nano.

[36] Haacke G (1976). New figure of merit for transparent conductors. J. Appl. Phys..

[37] De S, King PJ, Lyons PE, Khan U, Coleman JN (2010). Size Effects and the Problem with Percolation in Nanostructured Transparent Conductors. ACS Nano.

[38] Sorel S, Lyons PE, De S, Dickerson JC, Coleman JN (2012). The dependence of the optoelectrical properties of silver nanowire networks on nanowire length and diameter. Nanotechnology.

[39] Bae S (2010). Roll-to-roll production of 30-inch graphene films for transparent electrodes. Nat. Nanotechnol..

[40] Saha A, Jiang C, Martí AA (2014). Carbon nanotube networks on different platforms. Carbon.

[41] Deng B (2015). Roll-to-Roll Encapsulation of Metal Nanowires between Graphene and Plastic Substrate for High-Performance Flexible Transparent Electrodes. Nano. Lett..

