# Supplementary material for: Flash-induced nanowelding of silver nanowire networks for transparent stretchable electrochromic devices
Source: Sci Rep. 2018 Feb 9;8:2763. doi: 10.1038/s41598-018-20368-3 (PMC5807424; doi:10.1038/s41598-018-20368-3)
Supplement: Supplementary file 1 — Supplementary information [file 41598_2018_20368_MOESM1_ESM.doc]

Supplementary information

Flash Induced Nanowelding of Silver Nanowire Network for Transparent Stretchable Electrochromic device

**Chihak Lee^1,2^ , Youngsu Oh^1,2^ , In Seon Yoon^1,2^ , Sun Hong Kim^1^ , Byeong-Kwon Ju^2,*^ and Jae-Min Hong^1,3,4,*^**

^1^Photo-Electronic Hybrids Research Center, Korea Institute of Science and Technology (KIST), Seoul 02792, Republic of Korea

^2^Display and Nanosystem Laboratory, College of Engineering, Korea University, Seoul 02841, Republic of Korea

­^3^Division of Nano & Information Technology, KIST School, Korea University of Science and Technology, Seoul 02792, Republic of Korea.

^4^Institute of Advanced Composite Materials, Korea Institute of Science and Technology, Jeonbuk 55324, Republic of Korea

* [bkju@korea.ac.kr](mailto:bkju@korea.ac.kr), +82-2-3290-3671

* [jmhong@kist.re.kr](mailto:jmhong@kist.re.kr) +82-2-958-6601


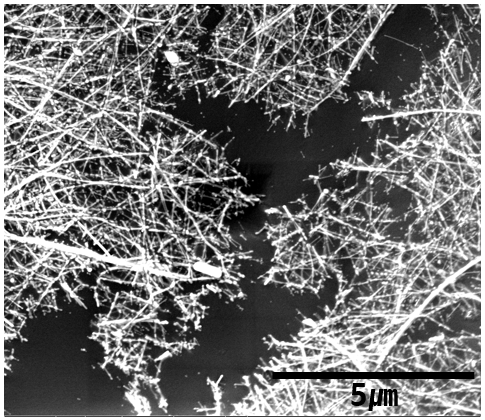


**Supplementary Figure S1**. SEM image of AgNW networks sample irradiated with flash light over 7J/cm^2^.


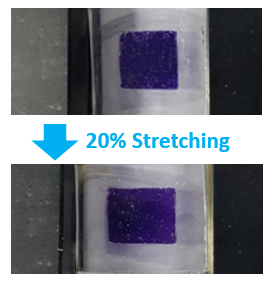


**Supplementary Figure S2.** ECD picture of initial state and after 20% increase.
